# An investigation of tendon strains in jersey finger injury load cases using a finite element neuromuscular human body model

**DOI:** 10.3389/fbioe.2023.1293705

**Published:** 2023-12-14

**Authors:** Lennart V. Nölle, Eduardo Herrera Alfaro, Oleksandr V. Martynenko, Syn Schmitt

**Affiliations:** ^1^ Institute for Modelling and Simulation of Biomechanical Systems, University of Stuttgart, Stuttgart, Germany; ^2^ Stuttgart Center for Simulation Science, University of Stuttgart, Stuttgart, Germany

**Keywords:** finite element analysis, injury criteria, jersey finger injury, muscle modelling, tendon strain injury

## Abstract

**Introduction:** A common hand injury in American football, rugby and basketball is the so-called jersey finger injury (JFI), in which an eccentric overextension of the distal interphalangeal joint leads to an avulsion of the connected musculus flexor digitorum profundus (FDP) tendon. In the field of automotive safety assessment, finite element (FE) neuromuscular human body models (NHBMs) have been validated and are employed to evaluate different injury types related to car crash scenarios. The goal of this study is to show, how such a model can be modified to assess JFIs by adapting the hand of an FE-NHBM for the computational analysis of tendon strains during a generalized JFI load case.

**Methods:** A jersey finger injury criterion (JFIC) covering the injury mechanisms of tendon straining and avulsion was defined based on biomechanical experiments found in the literature. The hand of the Total Human Model for Safety (THUMS) version 3.0 was combined with the musculature of THUMS version 5.03 to create a model with appropriate finger mobility. Muscle routing paths of FDP and musculus flexor digitorum superficialis (FDS) as well as tendon material parameters were optimized using literature data. A simplified JFI load case was simulated as the gripping of a cylindrical rod with finger flexor activation levels between 0% and 100%, which was then retracted with the velocity of a sprinting college football player to forcefully open the closed hand.

**Results:** The optimization of the muscle routing node positions and tendon material parameters yielded good results with minimum normalized mean absolute error values of 0.79% and 7.16% respectively. Tendon avulsion injuries were detected in the middle and little finger for muscle activation levels of 80% and above, while no tendon or muscle strain injuries of any kind occurred.

**Discussion:** The presented work outlines the steps necessary to adapt the hand model of a FE-NHBM for the assessment of JFIs using a newly defined injury criterion called the JFIC. The injury assessment results are in good agreement with documented JFI symptoms. At the same time, the need to rethink commonly asserted paradigms concerning the choice of muscle material parameters is highlighted.

## 1 Introduction

Many sporting activities put great stress on the upper appendages with around 25% of sports related injuries involving the hand or the wrist (Amadio, 1990; Rettig, 2003). A common hand injury in American football, rugby and basketball is the so-called jersey finger injury (JFI). This type of injury is caused by an eccentric overextension of the distal interphalangeal (DIP) joint, as can occur during the forceful release of one player’s grip on another player’s jersey or a finger getting caught on the rim of a basketball hoop, and leads to an avulsion of the connected *musculus flexor digitorum profundus* (FDP) tendon (Murphy and Mass, 2005; Gaston and Loeffler, 2015; Avery et al., 2016). This injury has been studied extensively in clinical studies, for example, in the work of Tempelaere et al. (Tempelaere et al., 2017), while computational investigations have thus far been focused on the modelling of general hand models (Joaquin et al., 2011), singular digits (Wu et al., 2008; Vigouroux et al., 2009; Fok and Chou, 2010; Wu et al., 2010) or the finger pulley system (Roloff et al., 2006; Vigouroux et al., 2008). In the field of automotive safety assessment, finite element (FE) neuromuscular human body models (NHBMs) created for the use with the FE-solver LS-DYNA (Ansys, Canonsburg, PA, United States) such as the Global Human Body Models Consortium (GHBMC) (Devane et al., 2019) or the Total Human Model for Safety (THUMS) (Kato et al., 2017; Kato et al., 2018) have been validated and are mainly employed to evaluate a host of different injury types related to car crash scenarios (GHBMC, 2016; Toyota Motor Corporation, and Toyota Central R&D Labs. Inc, 2021). Examples of these validation efforts are given by Kato et al. (Kato et al., 2018) where the THUMS model of version 6.0 was validated against several sets of test data derived from post-mortem human subjects (Cavanaugh et al., 1986; Cesari and Bouquet, 1990; Bolte et al., 2003; Foster et al., 2006; Rupp et al., 2008; Kroell et al., 2009; Shaw et al., 2009; Viano, 2009). The goal of this study is to show how a FE-NHBM can be modified to assess injuries not only found in car crashes, specific to the automotive domain, by adapting the hand of the THUMS AM50 occupant model of academic version 3.0 (Iwamoto et al., 2007) for the computational analysis of tendon strains during generalized JFI load cases. To this end, a Jersey Finger Injury Criterion (JFIC) covering the injury mechanisms of tendon straining and avulsion is first defined based on biomechanical experiments found in the literature. Next, FE-NHBM choice and necessary modification steps, including the routing and parameter tuning of newly introduced Hill-type muscles, will be outlined. Finally, a simulation study is performed to ensure both a sensible model behavior, and to tackle the question of how varying muscle activation levels and resulting maximum muscle forces impact the risk of sustaining a JFI in a representative injury scenario.

## 2 Materials and methods

### 2.1 Definition of a jersey finger injury criterion

JFI scenarios are characterized by the forced opening of an otherwise closed grip resulting in two distinct injury mechanisms. The main injury mechanism of the JFI is the avulsion of the FDP tendon caused by a hyperextension of the DIP joint (Gaston and Loeffler, 2015; Avery et al., 2016). Measurements of the forces necessary to induce such an injury are described in both the works of Holden and Northmore-Ball (Holden and Northmore-Ball, 1975) as well as those of Manske and Lesker (Manske and Lesker, 1978). To err on the side of caution, the lowest reported avulsion load of 10.8 kg (Manske and Lesker, 1978) was converted to Newtons and set as the resulting avulsion force threshold of 105.91 N. While not classically associated with the JFI, it is well known that eccentric muscle contraction can cause considerable damage to the affected tissue, with injuries ranging from minor strains to the complete rupture of the muscle-tendon-unit (MTU) (Noonan and Garrett, 1999; Maffulli, 2005). The eccentric lengthening of the finger flexor muscle groups was thus identified as a secondary injury mechanism to be represented in the JFIC. Studies of tendon material properties have shown that the severity of a sustained tendon strain injury can be linked to the deformation stages of the tendon’s stress-strain curve (Maffulli, 2005; Wang, 2006). Consequently, three distinct tendon strain injury thresholds were defined, with the minor injury threshold set at the start of the strain hardening region, the major injury threshold at the start of the necking region, and rupture threshold at the point of material failure. Tendon strains appropriate for the deformation regions of positional tendons as defined by Kaya et al. (Kaya et al., 2019) were derived from the literature (Maganaris et al., 2004; Wang, 2006; Stauber et al., 2019) and there thus used to define a secondary injury criterion called the Tendon Strain Injury Criterion (TSIC). A summary of the avulsion injury and TSIC threshold values, which together form the JFIC, is given in Table 1. The occurrence and severity of muscle strain injury was assessed using the Muscle Strain Injury Criterion (MSIC) analogously defined by Nölle et al. (Nölle et al., 2022b). An injury assessment using the JFIC and MSIC was performed for the FDP and the *musculus flexor digitorum superficialis* (FDS). All abbreviations used in the paper are listed in Supplementary Table S1.

**TABLE 1:** List of JFIC and TSIC threshold values.

Type of injury	Injury criterion	Threshold value	References
Minor Injury	JFIC, TSIC	4% strain	Maganaris et al. (2004), Wang (2006), Stauber et al. (2019)
Major Injury	JFIC, TSIC	8% strain	Maganaris et al. (2004), Wang (2006), Stauber et al. (2019)
Rupture	JFIC, TSIC	10% strain	Maganaris et al. (2004), Wang (2006), Stauber et al. (2019)
Avulsion	JFIC	105.91 N	Manske and Lesker (1978)

Abbreviations: JFIC, Jersey Finger Injury Criterion; TSIC, Tendon Strain Injury Criterion.

### 2.2 Model selection and modification

**FIGURE 1:**
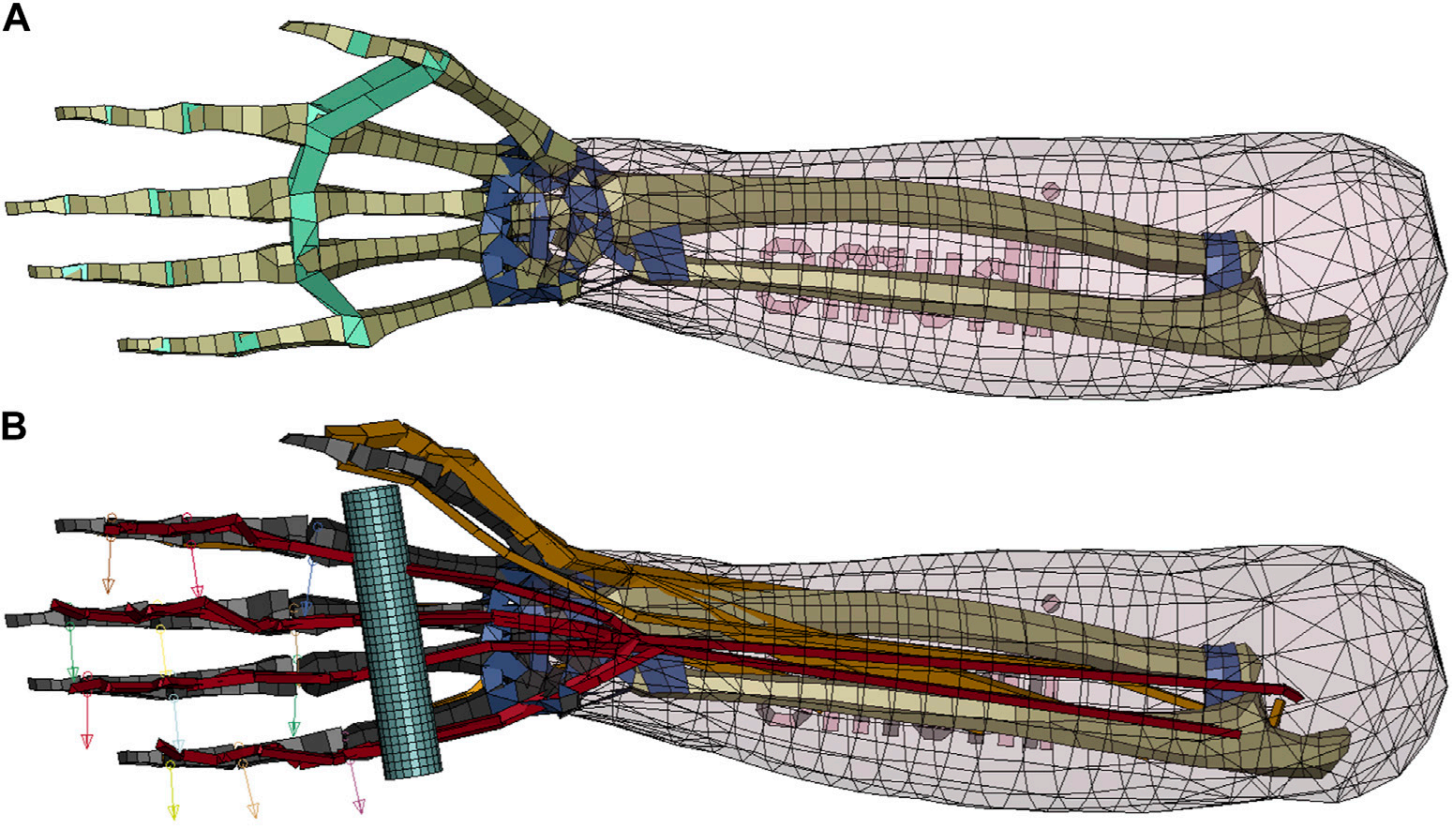
Comparison of the original and the modified THUMS version 3.0 hand models. **(A)** Original THUMS version 3.0 hand model, removed ligaments marked in green, kept ligaments marked in dark blue; **(B)** Modified THUMS version 3.0 hand model, rigid bones marked in black, manually routed muscles marked in orange, muscles with optimized routing marked in red, rod marked in light blue and revolute joint axes in the DIP, PIP and MCP joints marked with arrows.

The main selection criterion for the choice of an FE-NHBM was deemed to be the maximally achievable finger mobility, as simulating a gripping motion prior to the forced eccentric opening of the hand was considered to be essential for the reconstruction of JFI load cases. After evaluating the hand models of the THUMS AM50 occupant models version 3.0 (Iwamoto et al., 2007), version 4.1 (Shigeta et al., 2009), version 5.03 (Iwamoto and Nakahira, 2015) and version 6.1 (Kato et al., 2018), we arrived at the conclusion that no single model would be able to deliver a sufficient range of finger motion in their default states, as the mesh geometry and hand structures of all models limited the finger mobility to flexion movements only. Consequently, we decided to combine the properties of multiple models into one. THUMS version 3.0 was chosen as the base model because of the detailed modelling of the finger bone structure, while the muscle elements necessary for the generation of a flexion movement as well as the overall kinematic modelling approach were adopted from THUMS version 5.03. Both models were acquired under academic license from DYNAmore Gesellschaft für FEM Ingenieurdienstleistungen mbH, Stuttgart, Germany. All model modifications described in the following were performed on the right hand of the THUMS version 3.0. As a first modification step, the interphalangeal ligaments were removed to ensure appropriate finger movement capabilities. Second, the phalanges, metacarpals and carpals of the fingers were made rigid to allow for the insertion of kinematic revolute joints between the phalanges and metacarpals. To increase numerical stability, joint stiffness values taken from THUMS version 5.03 and joint range of motion limits described by Hirt et al. (Hirt, 2016) were implemented in a third step. Relevant flexor and extensor muscles of the hand were added based on the muscle modelling present in THUMS version 5.03 (Iwamoto and Nakahira, 2015) (Supplementary Table S2). Originally, these muscles are modelled using truss elements with the default LS-DYNA Hill-type muscle material *MAT_MUSCLE (LSTC, 2016b), while tendons are represented by seatbelt elements. To allow for a better assessment of tendon strain injury severity, *MAT_MUSCLE was replaced with a more biophysiological Hill-type muscle material developed by (Günther et al., 2007) and Haeufle et al. (Haeufle et al., 2014), which is available in LS-DYNA as a user-defined material named the extended Hill-type material (EHTM). The EHTM was initially implemented in LS-DYNA by Kleinbach et al. (Kleinbach et al., 2017) and updated to its most current version by Kleinbach et al. (Kleinbach, 2019), Martynenko et al. (Martynenko et al., 2023) and Wochner et al. (Wochner et al., 2022). Compared to *MAT_MUSCLE, the EHTM material has the additional benefit of including the tendon as a distinct element called the serial elastic element (SEE) (Haeufle et al., 2014), eliminating the need for combining muscle and seatbelt elements to form the MTU. However, one limitation of this modelling approach is that MTUs with a single muscle body and multiple connected tendons, as is the case for many muscles in the hand and lower arm, cannot be modelled as such but instead need be split up into discrete truss elements for each separate tendon path. For example, the FDP, a muscle with tendons reaching into fingers 2 to 5, was modelled as 4 parallel truss elements. A comparison of the original THUMS version 3.0 hand model and the modified version presented in this work is given in Figure 1.

### 2.3 Muscle routing and validation through moment arm optimization

The finger extensor muscle groups as well as the *musculus flexor pollicis longus* were manually routed using the via-point method (Delp et al., 1990; Hoy et al., 1990; Günther and Ruder, 2003) along anatomical landmarks, while special attention was given to the most injury-relevant flexors, FDP and FDS, whose routing paths across the DIP, proximal interphalangeal (PIP) and metacarpophalangeal (MCP) joint were additionally validated by adjusting them to fit moment arm curves compiled by Boots et al. (Boots et al., 2020) to ensure physiologically valid grip strength production and finger flexion mobility. Moment arms of FDP and FDS for the index finger were originally measured by Fowler et al. (Fowler et al., 2001), while data on the middle to ring finger were taken from Koh et al. (Koh et al., 2006). The nodal positions defining the muscle routing paths were optimized using the least-squares optimization functionality “lsqcurvefit” provided in the MATLAB R2022a Optimization Toolbox (Mathworks, Natick, MA, United States). The boundary conditions for the optimization were defined such that the routing nodes were placed on a plane which is normal to the revolute joint axes and intersects with the joint center. This condition was implemented to ensure that the force generated by the muscle elements could fully contribute to the resulting joint torque instead of partially dissipating by acting on a degree of freedom locked by the revolute joint. Additionally, nodes needed be placed on the medial palm side of the hand to avoid an overlap of the muscle trusses and the finger bones. The quality of the moment arm curves resulting from the optimized node placement was evaluated using the mean absolute error (Willmott and Matsuura, 2005) (Eq. (1)) normalized to the mean of the measured moment arm data (Eq. (2)).

$$MAE\left(\hat{y},y\right)=\sum_{i=1}^{n}\frac{\left|{\hat{y}}_{i}-y_{i}\right|}{n}$$ (1)

$$NMAE\left(\hat{y},y\right)=\frac{MAE\left(\hat{y},y\right)}{\frac{1}{n}\sum_{i=1}^{n}\left|y_{i}\right|}$$ (2)

where MAE is the mean absolute error, NMAE is the normalized mean absolute error, $\hat{y}$ is the predicted value, y is the measured value and n is the number of data points.

A total number of 20 moment arm curves, with the FDP spanning 4 digits over 3 joints and the FDS spanning 4 digits over 2 joints, were derived with the help of the routing path optimization. All optimized moment arm curves are shown in Supplementary Figures S1, S2. A detailed description of the moment arm optimization methodology is provided in Supplementary Chapter S3.

### 2.4 Tendon material parameter optimization

An assessment of JFI severity can only be reliably performed if the material parameters of the tendons are set within sensible bounds. On the one hand, overly compliant tendons would be a poor fit for the deformation characteristics of positional tendons and would limit finger movement capabilities, as the contractions of the muscles located in the lower arm could not be mechanically transferred to the fingers through the tendons but would instead be compensated for by an elongation of the tendon itself (Maffulli, 2005). On the other hand, overly stiff tendons would lead to an overestimation of MTU forces, which, in turn, would trigger the defined avulsion injury threshold (Table 1) erroneously. To avoid these issues, experimental data on the stress-strain characteristics of unembalmed human tendons collected by Benedict et al. (Benedict et al., 1968) were used for the manual tuning of the material parameters defining the tendon properties of the EHTM. These parameters are the force at the non-linear linear transition point ${∆F}_{SEE,0}$ , the relative stretch at non-linear-linear transition in $F_{SEE}\left(l_{SEE}\right)$ , $∆U_{SEE,nll}$ , and the relative stretch in the linear part for force increase of ${∆F}_{SEE,0}$ , $∆U_{SEE,l}$ . A graphical explanation of these parameters can be found in the work of Günther et al. (Günther et al., 2007). Data on the maximum isometric force $F_{\max}$ and the tendon cross-sectional area CSA , required for calculating equivalent stress-strain-curves with the EHTM, were taken from the works of Morales-Orcajo et al. (Morales-Orcajo et al., 2016) and Saraswat et al. (Saraswat et al., 2010). The curve fit quality for different parts of the stress-strain curve was assessed using two NMAE values, ${NMAE}_{3}$ covering the range of tendon strains between 0% and 3% and ${NMAE}_{5}$ for strains between 0% and 5% (Figure 3). Additionally, the Young’s modulus E was calculated from the linear parts of the resulting curves. All other non-generic muscle parameters of the EHTM were derived by converting the parameters of *MAT_MUSCLE with an adapted version of the method presented in the EHTM manual (Nölle et al., 2022a) and the work of Wochner et al. (Wochner et al., 2022). The aim of this method is to achieve a length equilibrium state during the initial simulation timestep in which the following condition holds true (Eq. (3)):

$$l_{MTU,i}=l_{CE,i}+l_{SEE,i}=l_{CE,opt}+l_{SEE,0}$$ (3)

where $l_{MTU,i}$ is the initial length of the MTU, $l_{CE,i}$ is the initial length of the EHTM contractile element CE, $l_{SEE,i}$ is the initial length of the EHTM serial elastic element SEE, $l_{CE,opt}$ is the optimal muscle fibre length and $l_{SEE,0}$ is the resting tendon length.

To account for the fact that the fingers of the THUMS version 3.0 model are straightened in its initial position and deviate from a relaxed hand position (Mount et al., 2003) in which the condition outlined in Eq. 3 could be assumed, we introduced an additional scaling factor $c_{e,f}$ to artificially shorten or elongate $l_{MTU,i}$ for the extensor and flexor muscle groups to represent their initially compressed or stretched state (Eq. (4)). The factor $c_{e,f}$ was manually set to achieve a MTU length equilibrium in which the hand of the model would close slightly on its own even when no external muscle activation was applied to reflect a neutral hand position (Mount et al., 2003).

$$c_{e,f}\;l_{MTU,i}=l_{CE,i}+l_{SEE,i};\;\;\;\;c_{e,f}=\left\{\begin{array}{c} 0.95,extensors\\ 1.05,flexors \end{array}\right.$$ (4)

where $c_{e,f}$ is the extensor-flexor scaling factor.

Additionally, the maximum isometric force of the muscles was scaled in cases where complex geometries of singular muscles had to be recreated with multiple parallel elements (Eq. (5)).

$$F_{\max,s}=\frac{F_{\max,T}}{n_{s}}$$ (5)

where $F_{\max,s}$ is the maximum isometric force of the muscle strand, $F_{\max,T}$ is the original $F_{\max}$ value found in THUMS version 5.03 and $n_{s}$ is the number of muscle strands.

A complete list of all EHTM material parameters used in the presented model can be found in Supplementary Tables S2, S3.

### 2.5 Simulated load case

A simplified JFI load case was defined by substituting the opponent’s jersey with a cylindrical rigid rod of 100 mm length and a diameter of 20 mm placed in the palm of the THUMS version 3.0 hand (Figure 1). Each JFI simulation included two consecutive stages (Supplementary Figure S3). In the first stage, covering the time interval of 0–100 ms, FDP and FDS were activated and given time to reach their flexion state in order to grab the rigid rod. The interaction between the hand and the rod was modelled using the an automatic surface-to-surface contact with static and dynamic friction values of 0.4 and 0.3 respectively (LSTC, 2016a). Additionally, a tied surface-to-surface contact with the same friction values (LSTC, 2016a) was activated after 80 ms to ensure that the rection forces of the surface-to-surface contact would not push the fingers apart to loosen the grip before the retraction of the rod. Once the rod gripping movement was completed, the ulna and radius were fully constrained in space to eliminate noise factors such as elbow extension or shoulder rotation during the rod pulling stage and to make sure that the entire stress caused by the JFI load case is placed on the finger flexors. In the second stage from $t_{R}$ = 100 ms to the simulation end time of 200 ms, the gripped rod was pulled out of the hand with a semi-instantaneous velocity of 11.615 m/s calculated using accelerations of sprinting college football players reported by (Brechue et al., 2010). The velocity and acceleration curves of the rod retraction are depicted in Supplementary Figure S4.

Eight JFI simulations at FDP and FDS muscle activation levels 0%, 20%, 40%, 60%, 70%, 80%, 90% and 100% were performed (simulations 1 to 8 in Table 3). An additional model check simulation (simulation 9 in Table 3) was done at 100%, in which the rod was not retracted, to ensure that the maximal muscle contraction alone did not cause injury by itself, for a total of 9 simulations. Muscle activation levels for all other muscles were kept at the minimum activation level defined by (Günther, 1997) to reflect their relaxed state. The effectiveness of the muscle material parameter dependent transfer between muscle activation a and the resulting MTU force $F_{MTU}$ was determined for FDP and FDS by calculating the muscle activation effectiveness $\eta_{a}$ (Eq. (6)).

$$\eta_{a}\left(a\right)=\frac{{\bar{F}}_{MTU,f,a}}{{\bar{F}}_{\max,f}}$$ (6)

where $\eta_{a}$ is the muscle activation effectiveness [0…100%] at muscle activation level a , ${\bar{F}}_{MTU,f,a}$ is the mean maximum muscle force of FDP and FDS for the time interval from 0 ms to 100 ms at muscle activation level a and ${\bar{F}}_{\max,f}$ is the mean maximum isometric muscle force of FDP and FDS.

All simulations were performed with a user-compiled double precision (DP) symmetric multiprocessing (SMP) version of LS-DYNA R9.3.1 (Ansys, Canonsburg, PA, United States) including EHTM version 3.2.04 (Nölle et al., 2022a). The simulations were run on a high-performance workstation equipped with an AMD Ryzen Threadripper 3990X 64-core processor (AMD, Santa Clara, CA, United States) using 32 SMP threads. The timestep size was automatically calculated with the timestep size for mass scaled solutions set to −6.000e-07 s. Simulation runtimes ranged between 4 h 31 m 25 s for simulation 5 and 7 h 12 m 58 s ± 3 m 16 s for all other simulations.

## 3 Results

### 3.1 Moment arm curve fit quality

**FIGURE 2:**
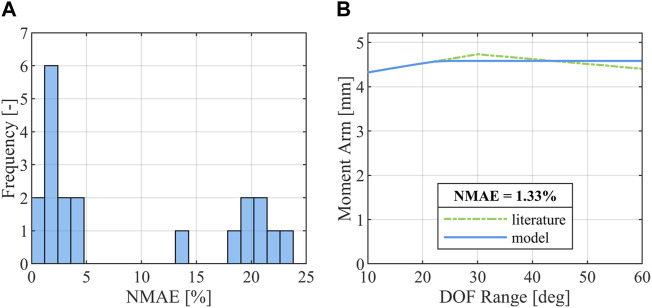
Results of the moment arm optimization. **(A)** Distribution of NMAE across all 20 optimized moment arm curves; **(B)** Comparison of moment arm curve reported in the literature (Fowler et al., 2001; Koh et al., 2006; Boots et al., 2020) and the model moment arm curve derived from optimization for FDP over the DIP joint in the index finger.

The optimization of the muscle routing node positions for FDP and FDS using moment arm data derived from the literature (Fowler et al., 2001; Koh et al., 2006; Boots et al., 2020) yielded good results with all moment arm curves showing NMAE values below 25% and 12 of the 20 optimized moment arm curves staying below NMAE = 5% (Figure 2A). An exemplary moment arm curve comparison for the FDP spanning the DIP joint is given in Figure 2B. A complete list of all moment arm curves can be found in Supplementary Figures S1, S2.

### 3.2 Optimized tendon material parameters

**FIGURE 3:**
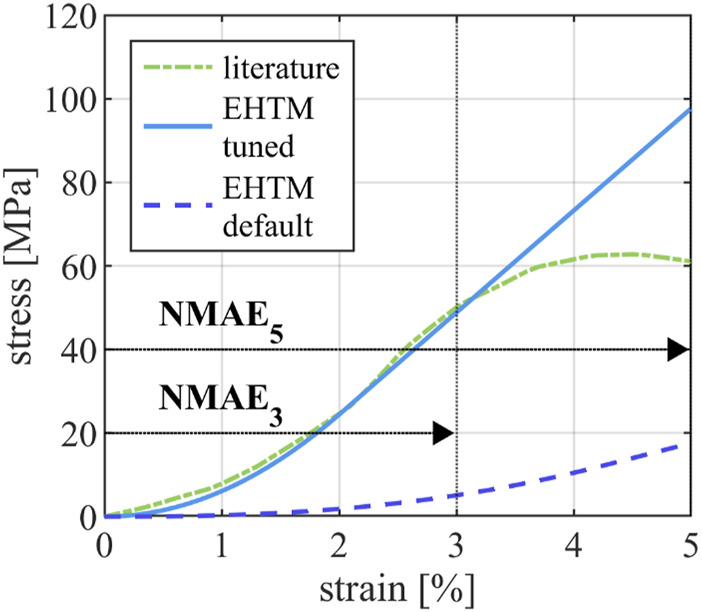
Comparison of the tendon stress-strain-curve reported in the literature (Benedict et al., 1968), the stress-strain curve of the EHTM tendon achieved through manual parameter fit and the stress-strain curve of the EHTM tendon using the default material parameters (Nölle et al., 2022a). Curve sections used for the calculation of ${NMAE}_{3}$ and ${NMAE}_{5}$ are indicated with arrows.

The manual tuning of EHTM tendon material properties to curves reported by Benedict et al. (Benedict et al., 1968) resulted in a greatly improved curve fit for tendon strains of up to 3%, with the tuned EHTM tendon material parameters scoring NMAE values of ${NMAE}_{3}$ = 7.16% compared to those of the default EHTM tendon parameters with ${NMAE}_{3}$ = 92.21% (Table 2). As such, the experimentally determined tendon stress-strain behavior is well represented by the tuned EHTM for this strain range. For higher tendon strains, the curve fit quality of the tuned EHTM decreases to ${NMAE}_{5}$ = 18.84% while still outperforming the default EHTM at ${NMAE}_{5}$ = 84.99%. The tuned tendon parameters are reflective of a much stiffer tendon than is commonly assumed for Hill-type muscles and differ greatly from the default material parameters of the EHTM (Nölle et al., 2022a) (Table 2), with a Young’s modulus of 2.4 GPa for both the tuned EHTM and the literature reference compared to E = 0.7 GPa for the default EHTM. The achieved curve fit is pictured in Figure 3.

**TABLE 2:** EHTM tendon material parameters and curve fit quality metrics.

Variable	Unit	EHTM tuned	EHTM default
${∆F}_{SEE,0}$	[N]	0.8 $F_{\max}$	0.4 $F_{\max}$
$∆U_{SEE,nll}$	[-]	0.02	0.0425
$∆U_{SEE,l}$	[-]	0.01	0.0170
${NMAE}_{3}$	[%]	7.16	92.21
${NMAE}_{5}$	[%]	18.84	84.99
E	[GPa]	2.4	0.7

Abbreviations: EHTM, extended Hill-type material.

### 3.3 Tendon and muscle strain injury assessment

**FIGURE 4:**
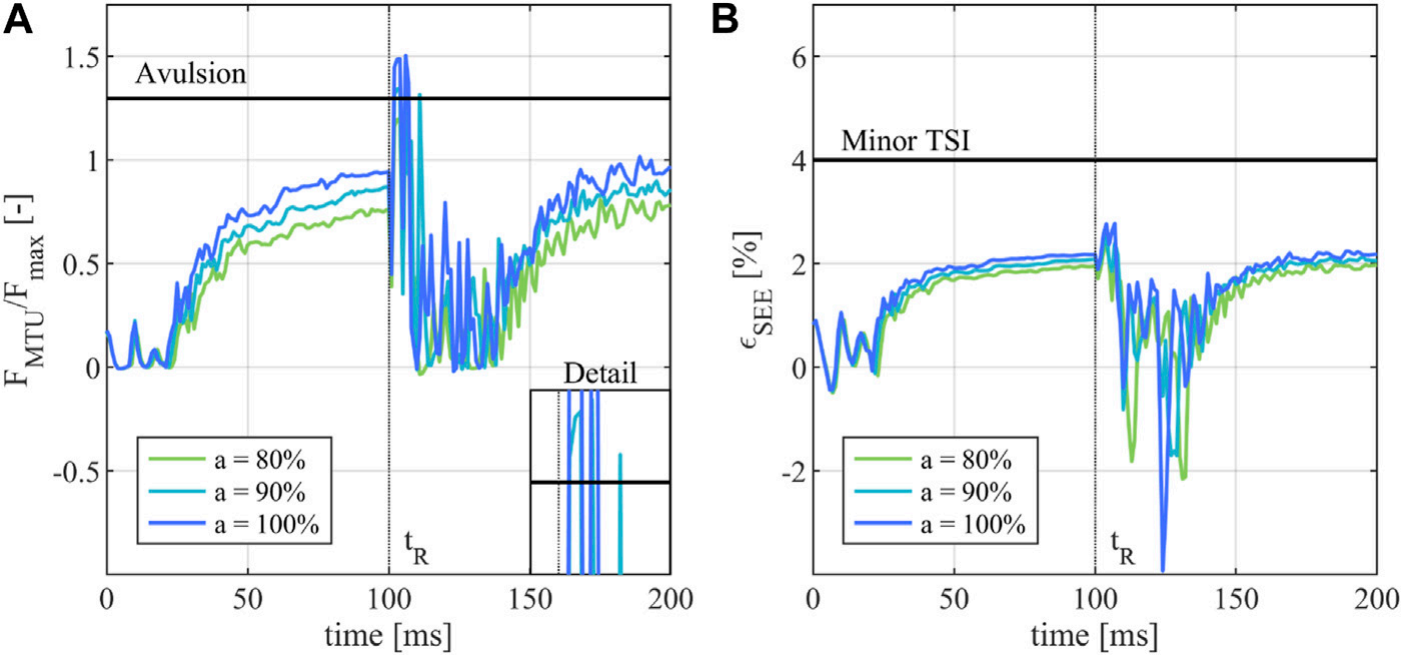
Injury assessment of the of the FDP in the middle finger during a JFI loading scenario. **(A)** Activity dependent normalized muscle force $F_{MTU}/F_{\max}$ compared to the JFIC avulsion injury threshold; **(B)** Resulting tendon strain $\varepsilon_{SEE}$ compared to the minor TSI threshold. The start of the rod retraction is denoted as $t_{R}$ .

The analysis of the MTU forces $F_{MTU}$ and the tendon strains $\varepsilon_{SEE}$ for simulations 1 to 9 was performed with two goals in mind. The first goal was to determine if the muscle material parameters of the modified THUMS version 3.0 hand model were set well and if the model itself yielded sensible injury assessment results by running the model check simulation 9. The results of model check simulation 9 (Table 3) show that the muscle parameters yield physiologically valid simulation results as the muscle activation of a = 100% is translated to an $\eta_{a}$ of 95.6% ± 1.3%, indicating that maximal muscle activation results in near maximal muscle force output. Additionally, the maximum muscle contraction on its own does not result in injury of any kind when assessed with both the MSIC and JFIC, confirming that the lower injury thresholds are set correctly in the sense that they do not register injuries during physiologically plausible muscle-driven gripping scenarios without external loads. Similarly, the results of simulations 1 to 5 (Table 3), covering the muscle activations of 0%–70% and $\eta_{a}$ values of 0.7% ± 0.6% to 68.0% ± 1.2%, show that the JFI loading scenario does not result in FDP or FDS injury, if insufficient muscle activation and thus reduced gripping force is applied. The first injury is detected in simulation 6 ( a = 80%, $\eta_{a}$ = 78.0 ± 1.5%) where the JFIC tendon avulsion injury threshold is crossed by the FDS of the middle finger after the rod retraction at $t_{R}$ (Table 3; Supplementary Figures S5, S6). The number of detected injuries further increases in Simulations 7 ( a = 90%, $\eta_{a}$ = 87.4 ± 1.5%) and 8 ( a = 100%, $\eta_{a}$ = 95.6 ± 1.3%) where additional avulsion injuries of the middle finger FDP (Table 3; Figure 4) and little finger FDP (Table 3; Supplementary Figures S5, S6) are detected. Overall, a clear relationship between muscle activity, resulting finger flexor muscle force and JFI occurrence can be established, with activation levels above 80% resulting in FDP and FDS avulsion injuries.

**TABLE 3:** List of simulations and injury assessment results.

Simulation No.	a [%]	$\eta_{a}$ ^a^ [%]	Rod retraction	No. of injuries	Type of injury	Injured MTU
1	0	0.7 ± 0.6	Yes	0	-	-
2	20	17.8 ± 1.8	Yes	0	-	-
3	40	39.4 ± 2.6	Yes	0	-	-
4	60	58.4 ± 1.8	Yes	0	-	-
5	70	68.0 ± 1.2	Yes	0	-	-
6	80	78.0 ± 1.5	Yes	1	Tendon Avulsion	FDS 3
7	90	87.4 ± 1.5	Yes	3	Tendon Avulsion	FDP 3, FDS 3, FDP 5
8	100	95.6 ± 1.3	Yes	3	Tendon Avulsion	FDP 3, FDS 3, FDP 5
9	100	95.6 ± 1.3	No	0	-	-

^a^ Data are represented as mean ± SD.

Abbreviations: FDP, musculus flexor digitorum profundus; FDS, musculus flexor digitorum superficialis; MTU, muscle-tendon-unit. Notes: Fingers are denoted according to the following numbering scheme: 1 = Thumb; 2 = Index Finger; 3 = Middle Finger; 4 = Ring Finger; 5 = Little Finger.

## 4 Discussion

The simulation-based reconstruction of sports injuries such as a JFI and the definition of criteria to assess such injuries is a challenging task, as numerous methodological approaches (Krosshaug et al., 2005) such as motion analysis, cadaver studies or athlete interviews yield insufficiently detailed information necessary for the one-to-one reconstruction of an injury. Additionally, only few instances of sports injuries during biomechanical experiments are described in the literature (Zernicke et al., 1977; Barone et al., 1999; Heiderscheit et al., 2005; Schache et al., 2009) with, to the authors’ knowledge, no documented case of an *in-vivo* JFI occurring in an experimental setup. Likewise, tendon strains and avulsion are not currently tracked in publicly available injury databases (Compton, 2002), further limiting the pool of load cases useable to define an injury criterion by conventional statistical means. The JFIC is thus based on the biomechanical properties of the human tendon instead of the statistical derivation of a risk index from documented injury cases. The tendon-strain-based injury thresholds of the TSIC were defined with the aim to represent the properties of positional tendons (Kaya et al., 2019), which are comparatively stiff and serve to transfer forces from the muscle to the bone with minimal force dissipation. As functional requirements influence the material properties of the tendon (Quigley et al., 2018), TSIC threshold values may need to be adjusted if applied to tendons which are known to be subjected to larger strain ranges such as the Achilles tendon. Similarly, the force-based JFIC avulsion threshold is only valid when applied to human finger flexor MTUs as the underlying experiments by Manske and Lesker (Manske and Lesker, 1978) were limited to the FDP tendon insertion only. Given that the JFIC was defined for the use with Hill-type muscle models such as the EHTM, tendon properties such as fatigue (Ker et al., 2000) or tendon creep (Maganaris, 2002; Maganaris et al., 2004) were not included in the definition of the injury criterion, as the used muscle model is not able to reflect these effects properly. Numerical instabilities common to Hill-type muscles as described by Yeo et al. (Yeo et al., 2023) were mitigated in this study by not routing muscles in series and by keeping muscle co-contraction levels to a minimum as the finger extensor muscles were only activated with a minimum physiological activity level (Günther, 1997). The modified THUMS version 3.0 hand model itself is well suited for the assessment of MTU forces and strains as is necessary for the proposed JFI assessment. Other anatomical structures such as the finger pulley system, the joint capsules or the soft tissues surrounding the phalanges are however not represented in the current model and offer room for further improvement of the model quality in future studies. Additionally, the previous validation efforts of both THUMS version 3.0 (Iwamoto et al., 2007) and 5.03 (Iwamoto and Nakahira, 2015) did not include specific validation cases for the lower arm or hand regions. The validity of the FE-NHBMs used in this study is thus transitively assumed, as the models performed well in the described whole-body validation cases, whose outcomes partly depend on the correct behavior of the upper extremities. The quality of the moment arm curve fit was determined using the NMAE as this metric provides an unweighted percentage result, meaning that the joint torque given as $F_{MTU}$ times moment arm length will deviate from the mean literature moment arm by the same NMAE percentage as the model moment arm itself. The achieved moment arm curve fit quality (Figure 2A) thus indicates that the majority of joint torques will be simulated with less than 5% error. The 8 worst moment arm curve fits ( NMAE between 13.87% and 23.72%) all occurred at the PIP joint, possibly indicating poor phalange geometry or inappropriately placed revolute joint axes in the PIP region. However, even the maximum NMAE of 23.72% (Supplementary Figure S1) is comparable to the standard deviations present in the reference literature data with Koh et al. (Koh et al., 2006) reporting FDP moment arm lengths over the MCP joint of 9.7 ± 2.0 mm equivalent to a deviation of 20.62%. While a good moment arm curve fit quality could thus be achieved, results might further be improved by implementing more advance routing methods such as the ellipse-based muscle routing proposed by Hammer et al. (Hammer et al., 2019) or by refining the positions of the finger joint axes. To the authors’ knowledge, no published data on the stress-strain behavior of unenbalmed human finger and lower arm tendons exist in the literature. Because of the similar anatomical structure, experimental data derived from the human lower extremities (Benedict et al., 1968) were used as a reference instead. The tuning of the tendon material parameters resulted in an EHTM tendon stress-strain behavior which is in good agreement with the literature data (Benedict et al., 1968) for tendon strains up to 3% ( ${NMAE}_{3}$ = 7.16%) but decreased in fit quality for larger strains ( ${NMAE}_{5}$ = 18.84%). This is due to the fact that the EHTM SEE only partially accounts for the deformation characteristics of a biological tendon as only the non-linear toe-region and an indefinitely continued linear elastic curve region are modelled (Günther et al., 2007). This means that tendon stress is overestimated for high tendon strains, which was deemed acceptable for the assessment of the presented JFI load case as the observed tendon strains never exceeded the minor TSIC threshold of 4% strain. Other muscle material models might however be needed to reliably determine tendon injury for load cases in which plastic tendon deformation is expected. Paradoxically, this overestimation of tendon forces in the EHTM could mitigate a common limitation of Hill-type muscles which are otherwise known to produce unphysiologically low forces in the eccentric muscle contraction range (Yeo et al., 2023). The Young’s modulus achieved through parameter optimization matched the literature reference (Benedict et al., 1968) exactly ( E = 2.4 GPa). This value is among the upper bound of vertebrate tendon stiffnesses described in the literature, where Young’s moduli ranging from 0.3 GPa (Maganaris et al., 2008) to 2.54 GPa (Ker et al., 1986) are reported. The modelled finger tendons can thus be described as very stiff, which is appropriate considering their mechanical function. The default EHTM tendon ( E = 0.7 GPa), while well suited to describe the passive mechanical properties of tendons found, for example, in the patella region (Wang, 2006), would thus not have been able to properly represent the finger tendons observed in this study. The simulated JFI load case is a simplified reconstruction of real-life injury scenarios, in which the jersey of an American football player has been replaced with a simple rod. This generalization is likely to reduce the detected injury severity, as the rod will always be cleanly released from the hand whereas the fabric of a jersey might catch on the fingers and subject the MTUs to even larger stresses. Future studies might focus on a more precise representation of JFI load cases. The results of the JFI assessment are congruent with JFIs as they are described in the literature (Tempelaere et al., 2017), given that the observed MTU forces under specific loads and muscle activations are high enough to register as tendon avulsions but do not trigger any of the other JFIC or MSIC thresholds. This means that the tendon and muscle body sustain no injury themselves but rather that the tendon insertion point is the weak link of the MTU chain. The detected dependency of injury occurrence and muscle activation can be explained by the synchronous rise in FDP and FDS muscle forces (Table 3) which place a larger stress on the tendon insertion before the eccentric rod retraction further increases the injury load. Translating this abstract description to a real-life injury scenario leads to the intuitive conclusion, that a stronger grip equates to an increased JFI severity. Contrary to JFI cases described in medical literature, JFI was only detected in the middle and little finger instead of the most injury-prone ring finger (Leddy and Packer, 1977; Manske and Lesker, 1978; Lunn and Lamb, 1984; Leddy, 1985; Bynum and Gilbert, 1988). This might be caused by several factors. First, the parallel muscle routing approach needed for the use of the EHTM, in which otherwise connected tendons operate as individual mechanical structures, might influence the loads acting on the now separated MTUs. Second, the simplifications of the load case and the model structure outlined above, while needed, will certainly impact the outcome of the simulations evaluated in the presented study. Finally, the muscle parameters which were adapted from the THUMS version 5.03 model might not be entirely representative of the average JFI patient. Muscle material parameters may vary greatly from person to person (Scovil and Ronsky, 2006) and the hand created by combining the hand geometry of THUMS version 3.0 and the musculature of THUMS version 5.03 might simply be representative of a person who belongs to the smaller group of people who suffer from JFI in less commonly injured digits (Murphy and Mass, 2005). In general, the topic of muscle material parameter choice should be considered if the MTU forces and strains are to be evaluated for injury assessment purposes, as they influence the detected injury severity most severely. Finetuning material parameters which are otherwise considered to be generic across all muscles, as is the case for the default EHTM tendon material parameters (Nölle et al., 2022a), may thus be needed to ensure a reliable injury detection.

### 4.1 Conclusion

The presented work outlines the steps necessary to successfully adapt the hand model of a FE-NHBMs for the assessment of JFIs using a newly defined injury criterion called the JFIC based on biomechanical data found in the literature, which, together with the previously established MSIC (Nölle et al., 2022b), forms a next step in creating a wholistic injury criterion for strain injuries of the MTU. The injury assessment results achieved with the JFIC are in good agreement with JFI symptoms as described in the medical field, showing a clear dependency between finger flexor activation, gripping force and JFI severity. At the same time, the need to rethink commonly asserted paradigms concerning the choice of muscle material parameters, in which only few parameters are assumed to be muscle specific, is highlighted, as material properties across all muscle structures need to be closely matched to the physiological demands of the muscle to ensure a reliable MTU injury assessment. Additionally, modelling choices and load case conditions should be representative of the real-life injury scenario, as to not subject the MTU to unrealistic loads. This work emphasizes the benefit of using neuromuscular human body models together with literature data and experiments to improve our understanding of how mechanical loads may cause tissue damage and thus, how to predict potential sources of injury. The authors hope to inspire further scientific cooperation between all fields of injury biomechanics with this interdisciplinary work of applying models commonly used in the automotive sector in a sports science context.

## Data Availability

The original contributions presented in the study are included in the article/Supplementary Material, further inquiries can be directed to the corresponding author.
