# Supplementary material for: An investigation of tendon strains in jersey finger injury load cases using a finite element neuromuscular human body model
Source: Front Bioeng Biotechnol. 2023 Dec 14;11:1293705. doi: 10.3389/fbioe.2023.1293705 (PMC10752991; doi:10.3389/fbioe.2023.1293705)
Supplement: Supplementary file 1 [file DataSheet1.pdf]

Supplementary Material

1 Supplementary Tables

Supplementary Table 1.
List of Abbreviations in alphabetical order.

Abbreviation	Meaning
DIP joint	Distal interphalangeal joint
DP	Double precision
EHTM	Extended Hill-type material
FDP	Musculus flexor digitorum profundus
FDS	Musculus flexor digitorum superficialis
FE	Finite element
GHBMC	Global Human Body Models Consortium
JFI	Jersey finger injury
JFIC	Jersey Finger Injury Criterion
MCP joint	Metacarpophalangeal joint
MSCI	Muscle Strain Injury Criterion
MTU	Muscle-tendon-unit
NHBM	Neuromuscular human body model
PIP joint	Proximal interphalangeal joint
SEE	Serial elastic element
SMP	Symmetric multiprocessing
THUMS	Total Human Model for Safety
TSIC	Tendon Strain Injury Criterion

Supplementary Table 2.
Specific muscle parameters of all implemented hand muscles.

**Muscle Name**	**Finger No.**	$\boldsymbol{F_{max}}$[1] **[N]**	$\boldsymbol{l_{CE,opt}}$ **[mm]**	$\boldsymbol{l_{SEE,0}}$ **[mm]**	$\boldsymbol{\Delta F_{SEE,0}}$ **[N]**	**PCSA**[1] **[mm²]**
M. flexor pollicis longus	1	77.2	288.07	43.38	61.76	348.0
M. extensor pollicis longus	1	39.5	52.83	216.19	31.60	110.0
M. extensor pollicis brevis	1	14.2	69.90	118.20	11.36	110.0
M. abductor pollicis longus	1	59.5	84.97	155.54	47.60	191.0
M. flexor digitorum superficialis	2	61.2	215.75	211.01	48.96	150.0
M. flexor digitorum profundus	2	68.3	170.43	253.24	54.64	225.0
M. extensor digitorum	2	18.3	79.91	367.06	14.64	107.5
M. extensor indicis	2	21.7	63.64	200.64	17.36	80.0
M. flexor digitorum superficialis	3	91.0	222.04	214.58	72.80	150.0
M. flexor digitorum profundus	3	81.7	183.71	272.88	65.36	225.0
M. extensor digitorum	3	35.3	80.37	373.78	28.24	107.5
M. flexor digitorum superficialis	4	57.9	207.07	189.67	46.32	150.0
M. flexor digitorum profundus	4	64.1	183.59	255.90	51.28	225.0
M. extensor digitorum	4	34.0	73.35	380.74	27.20	107.5
M. flexor digitorum superficialis	5	16.5	208.47	178.69	13.20	150.0
M. flexor digitorum profundus	5	79.7	175.53	227.54	63.76	225.0
M. extensor digitorum	5	13.1	77.95	356.19	10.48	107.5
M. extensor digiti minimi	5	25.3	61.50	291.18	20.24	90.0

Abbreviations: $\Delta F_{SEE,0}$ = Force at the nonlinear–linear transition in $F_{SEE}(l_{SEE})$; F_{max} = Maximum isometric force; $l_{CE,opt}$ = Optimal muscle fibre length; $l_{SEE,0}$ = Rest length of the serial elastic element; M. = Musculus; PCSA = Physiological cross-sectional area

Supplementary Table 3.
Generic muscle parameters of all implemented hand muscles.

Variable	Unit	Value	Description	Reference
q_0	[-]	0.005	Minimum value of muscle activity	[2]
c	[mol/L]	1.37e-4	Hatze constant c	[3]
η	[L/mol]	5.27e4	Hatze constant η	[3]
k	[-]	2.9	Hatze constant k	[3]
m	[1/s]	11.3	Hatze constant m	[3]
ΔW_{des}	[-]	0.45	Width of $F_{isom}(l_{CE})$ on descending limb	[4]
$\nu_{CE,des}$	[-]	1.5	Exponent of $F_{isom}(l_{CE})$ on descending limb	[5]
ΔW_{asc}	[-]	0.45	Width of $F_{isom}(l_{CE})$ on ascending limb	[4]
$\nu_{CE,asc}$	[-]	3	Exponent of $F_{isom}(l_{CE})$ on ascending limb	[5]
$A_{rel,0}$	[-]	0.2	Maximum value of A_{rel}	[2]
$B_{rel,0}$	[1/s]	2.0	Maximum value of B_{rel}	[2]
S_{ecc}	[-]	2.0	Step in inclination of $F_{CE}(\dot{l}_{CE} = 0)$ between eccentric and concentric force-velocity relations	[6]
F_{ecc}	[-]	1.5	Coordinate of pole in $l_{CE}(F_{CE})$ normalised to $F_{max} q F_{isom}(l_{CE})$ for $l_{CE} > 0$	[6]
$L_{PEE,0}$	[-]	0.95	Rest length of the PEE normalized to $l_{CE,opt}$	[2]
ν_{PEE}	[-]	2.5	Exponent of $F_{PEE}(l_{CE})$	[5]
F_{PEE}	[-]	2.0	Force of PEE if l_{CE} is stretched to ΔW_{des}	[5]
$\Delta U_{SEE,nll}$*	[-]	0.02	Relative stretch at non-linear-linear transition in $F_{SEE}(l_{SEE})$	-
$\Delta U_{SEE,l}$*	[-]	0.01	Relative stretch in linear part for force increase $\Delta F_{SEE,0}$	-
D_{SDE}	[-]	0.3	Dimensionless factor to scale $d_{SE,max}$	[5]
R_{SDE}	[-]	0.01	minimum value of d_{SE} normalised to $d_{SE,max}$	[5]

*Parameters determined in the presented work.

2 Supplementary Figures

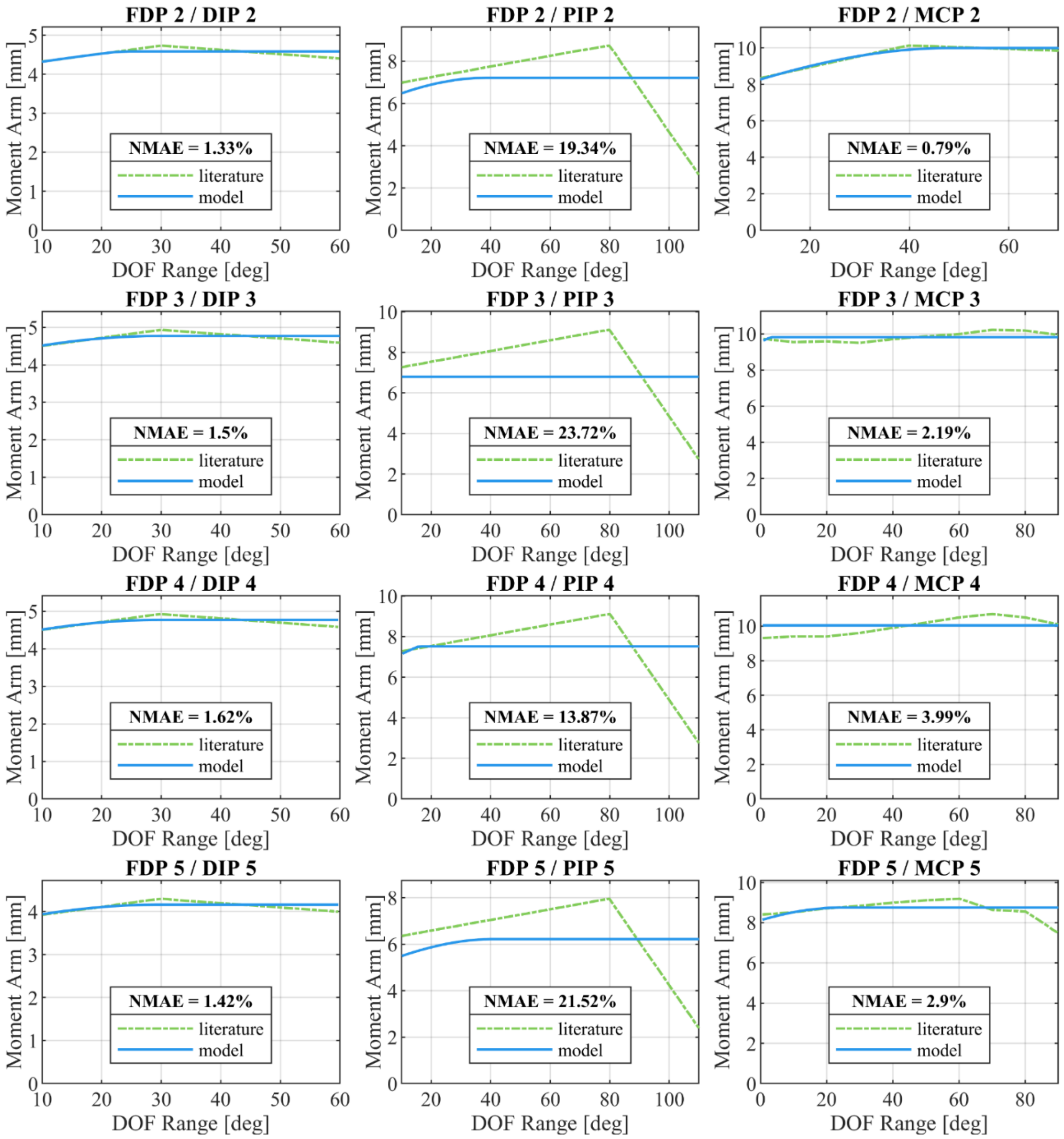

Supplementary Figure 1. Model moment arm curves of FDP spanning the DIP, PIP and MCP joints compared to moment arm curves reported in the literature[7–9].

Notes: Fingers are denoted according to the following numbering scheme:
1 = Thumb; 2 = Index Finger; 3 = Middle Finger; 4 = Ring Finger; 5 = Little Finger

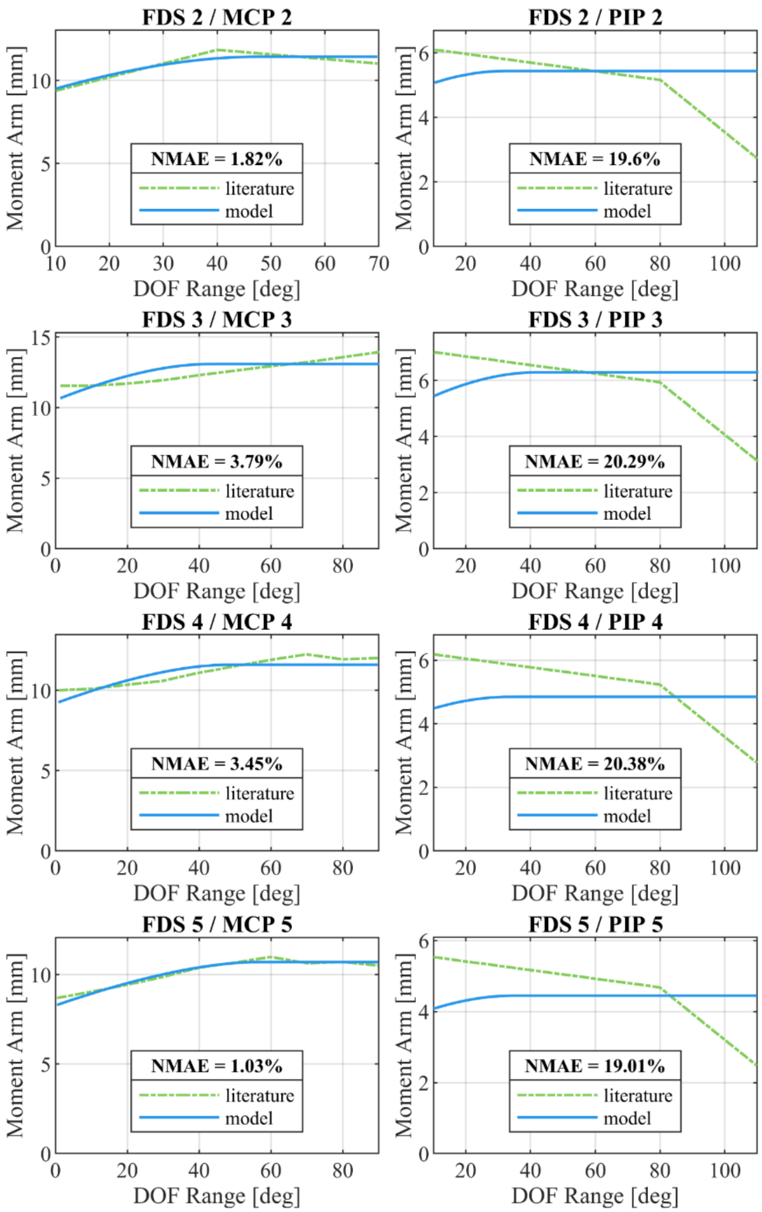

Supplementary Figure 2. Model moment arm curves of FDS spanning the PIP and MCP joints compared to moment arm curves reported in the literature[7–9].

Notes: Fingers are denoted according to the following numbering scheme:
1 = Thumb; 2 = Index Finger; 3 = Middle Finger; 4 = Ring Finger; 5 = Little Finger

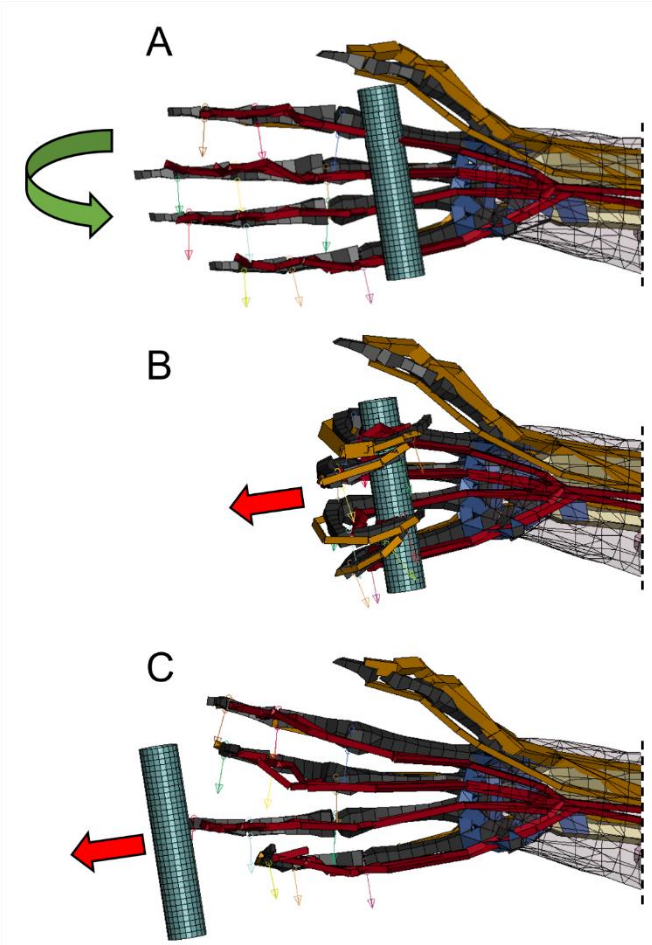

Supplementary Figure 3. Simplified Jersey Finger injury load case. A: FDP and FDS driven flexion to grip the rod (light blue) from 0 ms to t_R = 100 ms, finger flexion direction marked with a green arrow; B: Fully flexed hand position at t_R = 100 ms, rod retraction direction marked with a red arrow; C: Rod retraction from t_R = 100 ms to the simulation end time of 200 ms, rod retraction direction marked with a red arrow.

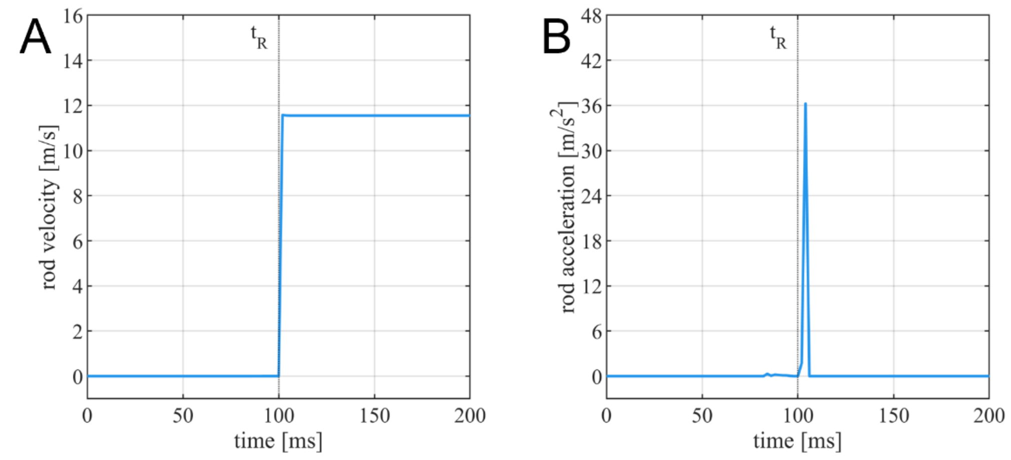

Supplementary Figure 4. Rod retraction characteristics. A: rod velocity curve; B: rod acceleration curve. The start of the rod retraction is denoted as t_R.

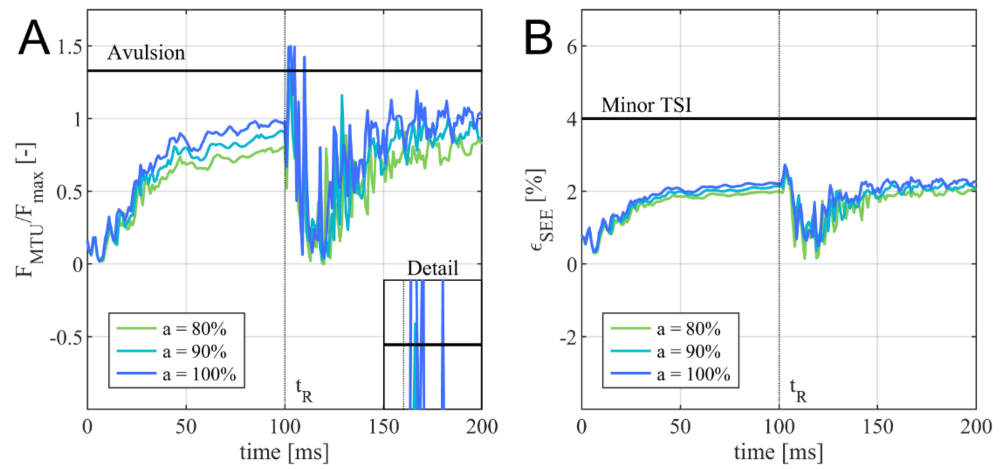

Supplementary Figure 5. Injury assessment of the of the FDP in the little finger during a JFI loading scenario. A: Activity dependent normalized muscle force F_{MTU}/F_{max} compared to the JFIC avulsion injury threshold; B: Resulting tendon strain ε_{SEE} compared to the minor TSI threshold. The start of the rod retraction is denoted as t_R.

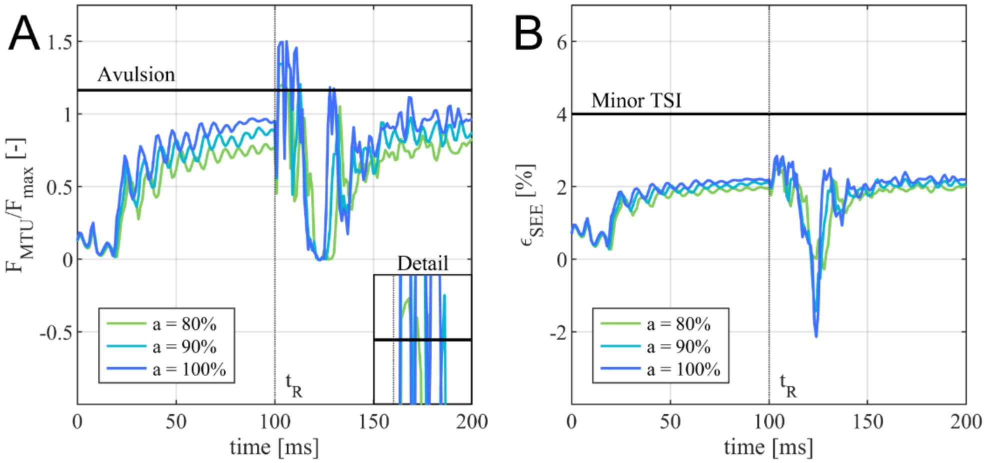

Supplementary Figure 6. Injury assessment of the FDS in the middle finger during a JFI loading scenario. A: Activity dependent normalized muscle force F_{MTU}/F_{max} compared to the JFIC avulsion injury threshold; B: Resulting tendon strain ε_{SEE} compared to the minor TSI threshold. The start of the rod retraction is denoted as t_R.

3 Muscle Routing Optimization Guide

The following guide will provide a detailed description of the optimization method used for placing the FDP and FDS routing nodes. To minimize the runtime, the optimization was not performed in the software environment of the FE solver LS-DYNA (Ansys, Canonsburg, PA, USA) but was instead done in MATLAB R2022a (Mathworks, Natick, MA, USA) using the least-squares optimization functionality “lsqcurvefit” provided in the Optimization Toolbox. Each muscle and each joint were tackled separately as to reduce the problem complexity. The optimization procedure will be explained for the example of the little finger FDP spanning the MCP joint. As such, “Body 1” refers to the little finger metacarpal, “Body 2” to the little finger proximal phalanx and the “Joint Axis” to the revolute axis of the little finger MCP joint (see Supplementary Figure 7).

1) Calculate relevant center points of Body 1 ($P_{B1,C}$, Eq. 1), Body 2 ($P_{B2,C}$, Eq. 2) and the Joint Axis (P_{JC}, Eq. 3).

$$P_{B1,C} = \left(P_{B1,1} + P_{B1,2}\right)/2 \quad \mathbf{1}$$

$$P_{B2,C} = \left(P_{B2,1} + P_{B2,2}\right)/2 \quad \mathbf{2}$$

$$P_{JC} = \left(P_{J1} + P_{J2}\right)/2 \quad \mathbf{3}$$

2) Calculate vectors that describe the orientations of Body 1 (v_{B1}, Eq. 4) and Body 2 (v_{B2}, Eq. 5) as well as the direction of the Joint Axis (v_J, Eq. 6).

$$v_{B1} = P_{JC} - P_{B1,C} \quad \mathbf{4}$$

$$v_{B2} = P_{JC} - P_{B2,C} \quad \mathbf{5}$$

$$v_J = P_{J2} - P_{J1} \quad \mathbf{6}$$

3) Calculate point necessary for routing node directional vectors. First, find the general form of plane A that intersects P_{JC} and has v_J as its normal vector (Eq. 7).

$$A\colon\ v_J \cdot P_{JC} = d \quad \mathbf{7}$$

Now, find the projection of $P_{B2,C}$, $P_{B2,CP}$, on A along v_J (Eq. 8, Eq. 9).

$$P_{B2,CP} = P_{B2,C} + \lambda\, v_J \quad \mathbf{8}$$

with

$$\lambda = \left(d - \left(v_J \cdot P_{B2,C}\right)\right)/\left(v_J \cdot v_J\right) \quad \mathbf{9}$$

4) Construct node placement vector v_1 between $P_{B2,CP}$ and P_{JC} whose length is determined by the proximal phalanx geometry (Eq. 10).

$$v_1 = P_{B2,CP} - P_{JC} \quad \mathbf{10}$$

5) Construct normalized node placement vector v_2 perpendicular to v_J and v_1 (Eq. 11).

$$v_2 = (v_1 \times v_J)/|v_1 \times v_J| \qquad \textbf{11}$$

Through these steps, two vectors v_1 and v_2 were defined, which guarantee that the routing nodes are placed on a plane which is normal to the revolute joint axes and intersects with the joint center. This ensures that the force generated by the muscle elements can fully contribute to the resulting joint torque.

6) Place muscle routing nodes $P_{R,i}$ starting from P_{JC} using linear combinations of v_1 and v_2. Nodes constrained to Body 1 are placed using Eq. 12 while nodes constrained to Body 2 are placed with Eq. 13.

$$P_{R,i} = P_{JC} + a_{B1,i}v_1 + b_i v_2 \qquad \textbf{12}$$

with $i = 1, \dots, n$; $a_{B1,i} \in [0.1, 0.5]$; $b_i \in [0.01, 30]$

$$P_{R,i} = P_{JC} + a_{B2,i}v_1 + b_i v_2 \qquad \textbf{13}$$

with $i = 1, \dots, n$; $a_{B2,i} \in [-0.5, -0.1]$; $b_i \in [0.01, 30]$

The limits defined for $a_{B1,i}$, $a_{B2,i}$ and b_i were set such that:

a) The nodes keep a minimum distance to the Joint Axis.
b) The nodes are placed on the medial palm side of the hand to avoid an overlap of the muscle trusses and the finger bones.

7) Rotate Body 2 and the routing nodes which are set as constrained to Body 2 around the Joint Axis while Body 1 is kept fixed. The joint angle is calculated as the angle between v_{B1} and v_{B2} using Eq. 14.

$$\varphi = acos((v_{B1} \cdot v_{B2})/(|v_{B1}|\,|v_{B2}|)) \qquad \textbf{14}$$

8) Calculate the moment arm as the shortest distance between the muscle beam defined through the nodes $P_{R,i}$.(see Supplementary Figure 8).
9) Plot the moment arm over φ and calculate the deviation from the reference moment arm using the NMAE (see Eq 1 and Eq 2 in the main manuscript).

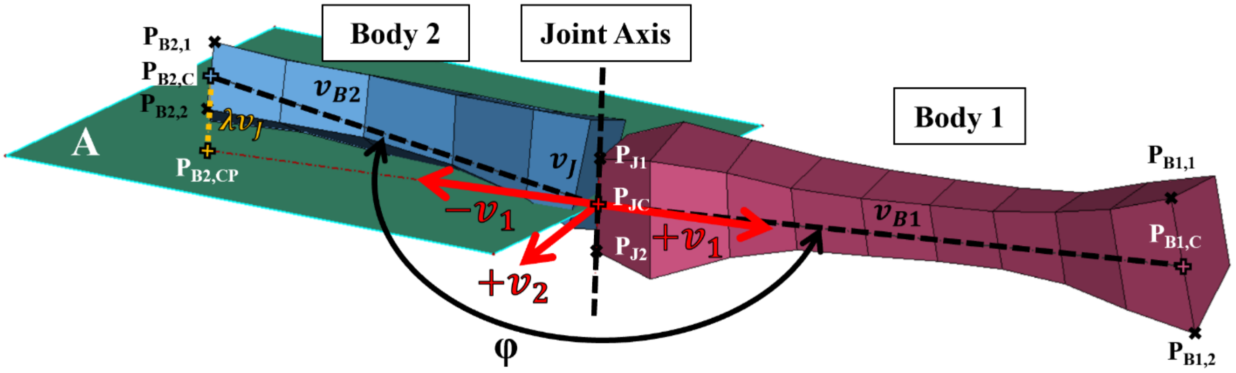

Supplementary Figure 7. Little finger MCP joint of the THUMS version 3.0 model[10] in LS-DYNA. The metacarpal (Body 1) is marked in magenta, the proximal phalanx (Body 2) is marked in blue. The point and vector descriptions correspond to point 1 to 6 in the "Muscle Routing Optimization Guide".

Given the information in steps 1 to 9, an optimization problem can be formulated, in which the placement of n routing nodes is adjusted through optimizing $a_{B1,i}$, $a_{B2,i}$ and b_i such that deviation between the resulting moment arm curve and the reference moment arm curve is minimized. This process was iteratively performed for $n = 3, \dots, 7$ and all possible combinations of splitting the n routing nodes between Body 1 and Body 2. For example, for $n = 4$, the following combinations were optimized for:

**Body 1**	**Body 2**
1 node	3 nodes
2 nodes	2 nodes
3 nodes	1 node

Additionally, two sets of optimizer settings for the MATLAB lsqcurvefit were applied to ensure that bad initial routing node choices would not impact the achieved curve fit:

Options 1:

```
options = optimoptions('lsqcurvefit', 'MaxFunctionEvaluations', 1e3,
'FunctionTolerance', 1e-6, 'FiniteDifferenceType', 'central', 'MaxIterations', 1e6,
'OptimalityTolerance', 1e-24, 'StepTolerance', 1e-9);
```

Options 2:

```
options = optimoptions('lsqcurvefit', 'MaxFunctionEvaluations', 1e3,
'FunctionTolerance', 1e-6, 'FiniteDifferenceType', 'forward', 'MaxIterations', 1e6,
'OptimalityTolerance', 1e-24, 'StepTolerance', 1e-9);
```

Considering all possible nodal combinations and the two sets of optimizer settings, 40 routing node configurations were optimized and evaluated for each muscle spanning each joint. From these 40 results, the one with the lowest NMAE was chosen to be included in the FE model.

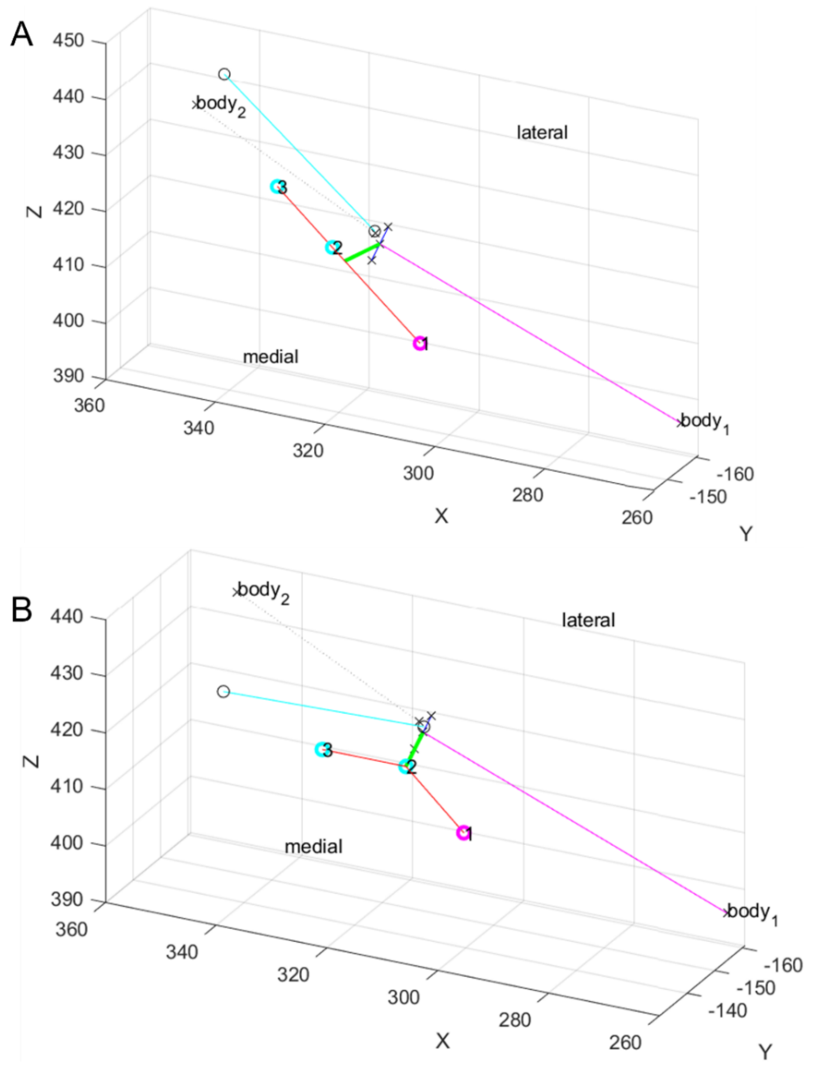

Supplementary Figure 8. Schematic representation of the little finger MCP joint in MATLAB. A: extended state of the MCP joint; B: flexed state of the MCP joint. The metacarpal (Body 1) is marked in magenta, the proximal phalanx (Body 2) is marked in blue. The three routing nodes are in the same color as the body which they are constrained to. The muscle routing path is marked in red, the resulting moment arm is marked in green.

4 References

1. Iwamoto M, Nakahira Y. Development and Validation of the Total HUman Model for Safety (THUMS) Version 5 Containing Multiple 1D Muscles for Estimating Occupant Motions with Muscle Activation During Side Impacts. In: *SAE Technical Paper Series:* SAE International400 Commonwealth Drive, Warrendale, PA, United States; 2015, doi:10.4271/2015-22-0003.
2. Günther M. *Computersimulationen zur Synthetisierung des muskulär erzeugten menschlichen Gehens unter Verwendung eines biomechanischen Mehrkörpermodells*; 1997.
3. Kistemaker DA, van Soest AJ, Bobbert MF. Is equilibrium point control feasible for fast goal-directed single-joint movements? *Journal of neurophysiology* 2006;**95**(5):2898–912, doi:10.1152/jn.00983.2005.
4. Bayer A, Schmitt S, Günther M, Haeufle DFB. The influence of biophysical muscle properties on simulating fast human arm movements. *Comput Methods Biomech Biomed Engin* 2017;**20**(8):803–21, doi:10.1080/10255842.2017.1293663.
5. Mörl F, Siebert T, Schmitt S, Blickhan R, Günther M. Electro-Mechanical Delay In Hill-Type Muscle Models. *J. Mech. Med. Biol.* 2012;**12**(05):1250085, doi:10.1142/S0219519412500856.
6. van Soest AJ, Bobbert MF. The contribution of muscle properties in the control of explosive movements. *Biological cybernetics* 1993;**69**(3):195–204, doi:10.1007/BF00198959.
7. Boots MT, Hardesty R, Sobinov A, Gritsenko V, Collinger JL, Fisher LE, et al. *Functional and Structural Moment Arm Validation for Musculoskeletal Models: A Study of the Human Forearm and Hand*; 2020, doi:10.1101/2020.05.29.124644.
8. Fowler N, Nicol A, Condon B, Hadley D. Method of determination of three dimensional index finger moment arms and tendon lines of action using high resolution MRI scans. *J Biomech* 2001;**34**(6):791–7, doi:10.1016/S0021-9290(01)00021-5.
9. Koh S, Buford WL, Andersen CR, Viegas SF. Intrinsic muscle contribution to the metacarpophalangeal joint flexion moment of the middle, ring, and small fingers. *J Hand Surg Am* 2006;**31**(7):1111–7, doi:10.1016/j.jhsa.2006.03.003.
10. Iwamoto M, Nakahira Y, Tamura A, Kimpara H, Watanabe I, Miki K. Development of advanced human models in THUMS. In: *Proc. 6th European LS-DYNA Users' Conference*; 2007, p. 47–56.
